# *Enterobacter cloacae* septicemia in a triple-cannula extracorporeal membrane oxygenation circulatory support treated with Seraph-100 Microbind affinity blood filter

**DOI:** 10.3325/cmj.2023.64.284

**Published:** 2023-08

**Authors:** Marin Pavlov, Tomislava Bodrožić Džakić Poljak, Nikola Pavlović, Sandra Šestan Crnek, Davor Barić, Igor Rudež

**Affiliations:** 1 Department of Cardiology, Dubrava University Hospital, Zagreb, Croatia; 2 Department of Clinical Microbiology and Hospital Infections, Dubrava University Hospital, Zagreb, Croatia; 3 Department of Cardiac and Transplant Surgery, Dubrava University Hospital, Zagreb, Croatia; 4 University of Zagreb School of Medicine, Zagreb, Croatia; Pavlov et al: Seraph-100 for extracorporeal membrane oxygenation related bloodstream infection

## Abstract

Bloodstream infections (BSI) are frequently encountered during extracorporeal membrane oxygenation (ECMO) support. Once septicemia is observed, treatment should be rapid, adequate, and multifaceted, particularly in advanced ECMO configurations. We report on a case of a 60-year-old male patient with acute-on-chronic heart failure due to ischemic cardiomyopathy. The treatment was complicated by cardiogenic shock requiring veno-arterial ECMO support, and, due to persistent pulmonary congestion, an upgrade with an additional left-atrial drainage cannula. After seven days of ECMO support, septicemia with shock ensued. *Ex iuvantibus* antibiotic treatment was started promptly. We wanted to minimize the likelihood of bacterial biofilm build-up requiring an exchange of the ECMO circuit and cannula, which was expected to be challenging. Therefore, we added a Seraph-100 Microbind affinity blood filter (providing blood purification with the potential for rapid bacterial clearance) to the ECMO circuit. Initial blood cultures tested positive for *Enterobacter cloacae*. Following a course of Seraph-100 treatment, bacteremia, septicemia, and shock resolved. There was no need for a circuit or cannula exchange. The additional eleven days of ECMO support were uneventful. The patient was successfully bridged to long-term mechanical circulatory support. We believe that the synergistic effect of early implementation of both broad-spectrum antibiotic treatment and blood purification with the potential for rapid bacterial clearance (such as the one provided with the Seraph-100 Microbind affinity blood filter) is crucial in BSI in patients receiving advanced ECMO.

Bloodstream infections (BSI) present a major concern during mechanical circulatory support. For example, BSI delay definite treatment in patients requiring veno-arterial (VA) extracorporeal membrane oxygenation (ECMO) as a bridge to surgery. As circuit colonization may lead to an unremitting course of BSI (1), aggressive infection control is of foremost importance. We present a case of *Enterobacter cloacae* septicemia in a patient with triple-cannula circulatory VA ECMO support treated with Seraph-100 Microbind affinity blood filter. Several reports propose the use of Seraph-100 in ECMO patients, but we found no report on *Enterobacter cloacae* septicemia treated with Seraph-100. Furthermore, triple cannula VA ECMO configuration is rarely employed, and Seraph-100 is predominantly used attached to the hemodialysis machines rather than to ECMO.

## Case report

**Figure 1:**
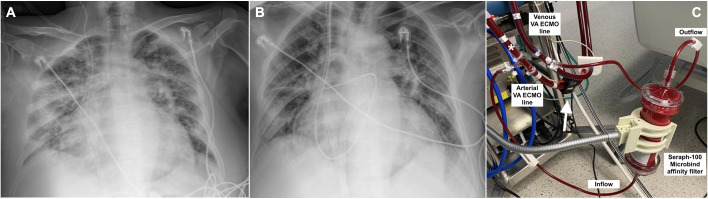
(**A**) The initial veno-arterial extracorporeal membrane oxygenation (ECMO) support configuration; pulmonary congestion. (**B**) An upgrade with a left atrial drainage cannula inserted via the interatrial septum; regression of pulmonary congestion. (**C**) Connecting the Seraph-100 Microbind affinity blood filter to a running ECMO circuit, as described by Ronco et al (12) requires the addition of a sideport with a three-way stopcock into both the arterial and venous tubing. After priming the filter, the ¼” tubing (included in the filter set) is connected to the sideports, thus establishing the flow through the filter. The possibility of flow regulation in the ECMO setup is limited and depends on the set ECMO flow. The flow through the filter (250 mL/min in this case) can be determined with dedicated flowmeters (not included in the Maquet Cardiohelp system). Additionally, it can be estimated by subtracting the arterial flow measured downstream from the filter inflow limb (arrow – actual arterial flow delivered to the patient) from the total arterial ECMO output (measured at the level of the asterisk). Alternatively, the filter can be added to the ECMO circuit via a renal replacement therapy device, which assures improved handling of the filter flow.

**Figure 2:**
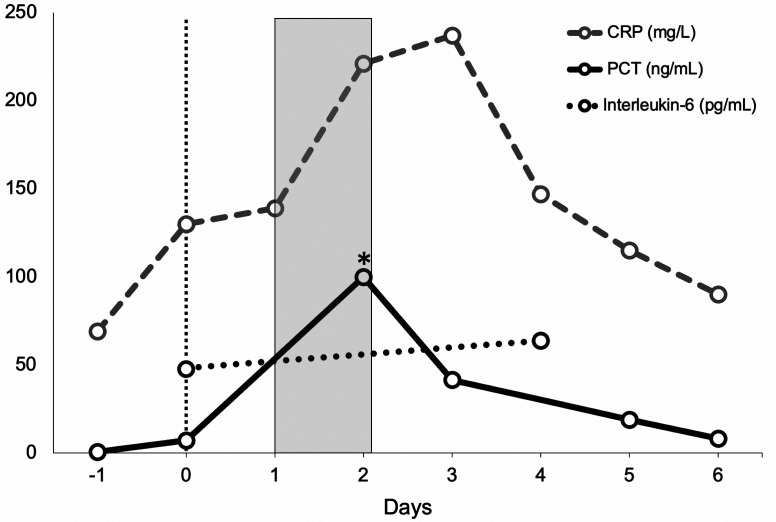
C-reactive protein (CRP) and procalcitonin (PCT) peaked following the Seraph-100 Microbind affinity blood filter treatment. Interleukin-6 values were not markedly elevated. The asterisk denotes the value above the upper limit of detection (>100.0 ng/mL)

**Figure 3:**
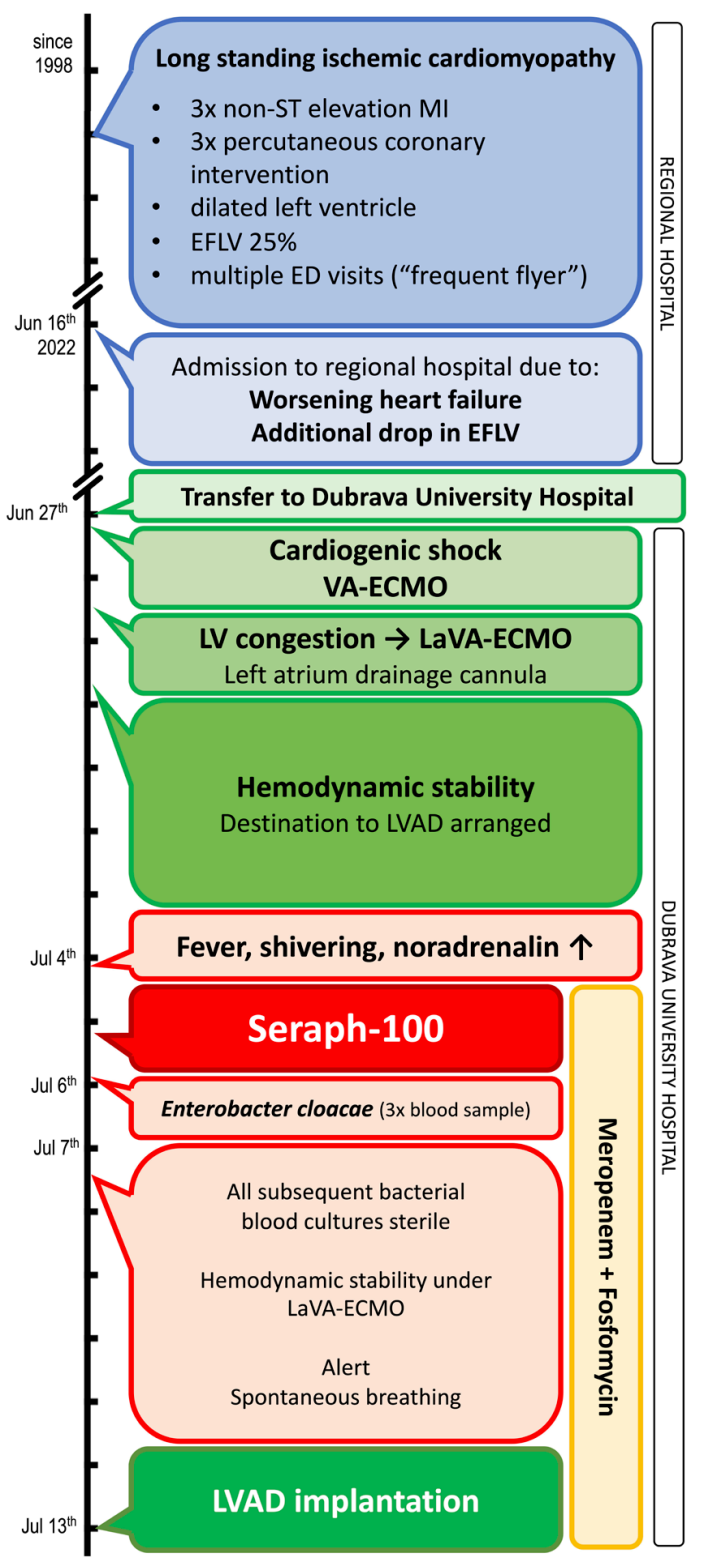
The timeline of events and procedures. MI - myocardial infarction; EFLV - ejection fraction of the left ventricle; ED - emergency department; VA-ECMO - veno-arterial extracorporeal membrane oxygenation; LVAD - left ventricular assist device.

A 60-year-old male patient was referred to our center from a regional hospital for pre-transplant work-up. The patient had been treated for biventricular heart failure due to ischemic cardiomyopathy for 25 years. The most recent echocardiography revealed a dilated left ventricle (LV) (67 mm) with ejection fraction of 25%, dilated right ventricle (55 mm) with diminished contractility (tricuspid annular plane excursion 11 mm), moderate mitral regurgitation, and a competent aortic valve. He had been frequently treated with intravenous loop diuretics for congestion in a local emergency department. During the last hospital admission, an additional drop in LV systolic function was observed, necessitating advanced heart failure treatment. On admission to our center, the patient was alert, dyspneic, and tachypneic. Low blood pressure (67/40 mm Hg) and sinus tachycardia (107/min) were recorded. Emergency echocardiography revealed an LV ejection fraction of 14%. Since hypotension did not improve with dobutamine (up to 20 µg/kg/min) and norepinephrine (up to 0.5 µg/kg/min), and arterial lactate levels suggested early shock stages (3.5 mmol/L), urgent VA ECMO with percutaneous cannulation was arranged. Venous (23F 55 cm) and arterial (21F 23 cm) sheaths were introduced in the right common femoral vein and the left common femoral artery, respectively. A circuit was established by using Cardiohelp with HLS Set Advanced 7.0 (Maquet, Rastatt, Germany). An initial support of 4.0 L/min ensured adequate blood pressure and perfusion. However, LV dilatation and lung congestion were observed (Figure 1A), refractory to inotrope dose adjustments. Thus, left atrial venting with a 23F TandemHeart transseptal cannula (Cardiac Assist, Inc., Pittsburgh, PA, USA) (left common femoral vein approach) following intracardiac ultrasound-guided atrial septal puncture was performed. This ensured adequate LV decongestion while preserving optimal perfusion (Figure 1B). Multiple weaning attempts were ineffective due to remitting hypotension. At this stage, left ventricular assist device implantation was arranged. On day 7 of VA ECMO support, the patient experienced extensive shivering and elevated body temperature (38.3 °C). Laboratory workup revealed increased C-reactive protein and procalcitonin levels (Figure 2). Norepinephrine up to 0.2 µg/kg/min was required to maintain adequate mean arterial blood pressure. During febrile events, four sets of blood cultures were collected. On the same day, three sets tested positive for *Enterobacter cloacae*. The isolate was susceptible to meropenem, imipenem, gentamicin, amikacin, ciprofloxacin, and trimethoprim-sulfamethoxazole. Extended-spectrum beta-lactamase and carbapenemase production was not detected. Empirical therapy with meropenem 2 g iv over three-hour infusion three times a day and fosfomycin 8 g three times a day was initiated. In addition, at antibiotic treatment initiation (before microorganism detection), a Seraph-100 Microbind affinity blood filter (Exthera Medical Corporation, Martinez, CA, USA) was added to the circuit for 26 h (Figure 1C). After 24 h of blood purification, the fever resolved, norepinephrine was stopped, and inflammatory markers peaked (Figure 2). Laboratory findings were not suggestive of hemolysis. Multiple control blood cultures remained negative. Antibiotic therapy was resumed during VA ECMO support (additional 11 days). Throughout the treatment, the patient was alert. Mechanical ventilation was not required. The patient eventually underwent left ventricular assist device implantation (Figure 3).

## Discussion

Members of the *Enterobacter cloacae* complex are facultative anaerobic Gram-negative bacilli that are often a part of the commensal intestinal flora in humans. As opportunistic pathogens, they cause serious nosocomial infections and outbreaks. The rates of *Enterobacter cloacae*-positive isolates in patients treated with ECMO range from 2.1% to 9.43% (2,3) out of all positive samples. Bacteremia due to *Enterobacter cloacae* occurs in an earlier phase (less than 7 days) after cannulation (4). In our patient, the first bacteremia occurred seven days after cannulation.

Whether BSI affects the outcome of patients on ECMO support is still a matter of debate (5). Nevertheless, in our case BSI delayed surgical treatment. The triple-cannula configuration would have rendered cannula and ECMO circuit exchange exceptionally demanding, which is why rapid and complete BSI resolution was of utmost importance. The therapy of choice in these cases is a combined, high-dose antibiotic treatment, preferably with continuous rather than bolus dosing. In ECMO patients with BSI, the appropriate antibiotic should be able to penetrate bacterial biofilms, a feature ascribed to fosfomycin (6).

Seraph-100 Microbind affinity blood filter is a single-use, disposable column, made by polyethylene beads with end point-attached heparin at its surface (7). Heparin mimics negatively charged heparan sulfates on cell surface, thus providing adsorption and up to a 90% reduction in bloodstream pathogens during a single treatment. Several reports (8,9) propose the use of Seraph-100 in ECMO patients. In a report of *Staphylococcus aureus* septicemia treated with Seraph-100 blood filter, bacterial clearance was determined by taking blood samples from the inflow and outflow arm of the filter at three time points (10). Outflow samples were sterile immediately (the first sample taken after 5 min of treatment), while inflow samples became sterile at the third sampling (10 hours into the treatment). In our case, all cultures were taken from venous blood samples, a procedure preventing an exact estimation of the bacterial clearance provided by the blood filter. However, rapid resolution of bacteremia was suggested by the clinical course, laboratory findings, and control cultures. Despite high levels of C-reactive protein and procalcitonin, interleukin-6 levels were only mildly elevated both before and after the treatment, indicating that blood purification was more associated with bacterial than with proinflammatory cytokines clearance.

Connecting the filter to the running ECMO circuit is undemanding. The required filter flow (100-350 mL/min) elicits acceptable level of recirculation. The amount of antibiotic extraction is possibly negligible, although further studies on this issue are required (11). In this case, it is difficult to determine the exact individual contributions of antibiotic treatment and blood purification to septicemia resolution. In our opinion, what played a major role was the synergistic effect of early implementation of the two strategies. There is a need for further studies, preferentially randomized controlled trials, to assess the effect of the Seraph-100 Microbind affinity blood filter on the clinical outcomes of BSI in ECMO patients. Until such studies are available, we suggest using the described combined approach in this setting.
